# Down-regulating NEAT1 inhibited the viability and vasculogenic mimicry formation of sinonasal squamous cell carcinoma cells via miR-195-5p/VEGFA axis

**DOI:** 10.1042/BSR20201373

**Published:** 2020-11-19

**Authors:** Honglue Lu, Fei Kang

**Affiliations:** Department of Otolaryngology, Affiliated Hospital of Chengde Medical University, Chengde, Hebei 067000, China

**Keywords:** miR-195-5p, nuclear enriched abundant transcript 1, sinonasal squamous cell carcinoma, vascular endothelial growth factor A, vasculogenic mimicry

## Abstract

The role of long non-coding RNA nuclear-enriched abundant transcript 1 (lncRNA NEAT1) in sinonasal squamous cell carcinoma (SNSCC) remained obscure. Target genes and potential binding sites of NEAT1, microRNA (miR)-195-5p and VEGFA were predicted using StarBase and TargetScan, and confirmed by dual-luciferase reporter assay. Quantitative real-time polymerase chain reaction (qRT-PCR) was performed to detect the expressions of NEAT1, vascular endothelial growth factor A (VEGFA) and miR-195-5p. Pearson’s correlation analysis of NEAT1, miR-195-5p and VEGFA was conducted. Cell viability, apoptosis and tube formation capability were assessed by MTT assay, flow cytometry and capillary-like tube formation assay, respectively. Expressions of VEGFA and proteins related to the phosphatidylinositide 3-kinase/Protein Kinase B (PI3K/AKT) pathway were measured by Western blot. In SNSCC tissues and cells, the expressions of NEAT1 and VEGFA were up-regulated while the expression of miR-195-5p was down-regulated, and NEAT1 was negatively correlated with miR-195-5p yet positively correlated with VEGFA. Overexpressed VEGFA promoted the viability and capillary-like tube formation of SNSCC cells yet suppressed their apoptosis, while silencing VEGFA led to the opposite results. MiR-195-5p could bind to NEAT1, and down-regulating miR-195-5p reversed the effects of silencing NEAT1 on the expressions of NEAT1 and miR-195-5p, cell viability, apoptosis and capillary-like tube formation as well as PI3K/AKT pathway activation. VEGFA was the target of miR-195-5p, and overexpressed VEGFA reversed the effects of miR-195-5p. Down-regulating NEAT1 inhibited the viability and vasculogenic mimicry formation of SNSCC cells yet promoted their apoptosis via the miR-195-5p/VEGFA axis, providing a possible therapeutic target for SNSCC treatment.

## Introduction

Sinonasal malignancies are rarely occurring tumors which account for less than 3% of overall head and neck cancers [1]. Squamous cell carcinoma is the most common type of sinonasal malignancy, occupying up to 75% of all sinonasal malignancies [2]. Sinonasal malignancies are categorized as aggressive tumors because patients, who remain asymptomatic before diagnosis, are generally diagnosed at an advanced stage when the tumor has grown big enough to manifest symptoms. [3]. Though remarkable progress has been made in surgery and therapy, the prognosis of patients with sinonasal squamous cell carcinoma (SNSCC) remained poor [4]. Therefore, it is of great urgency and significance to further discover the molecular mechanisms related to SNSCC development and progression.

Long non-coding RNAs (lncRNAs) are a family of transcripts which are longer than 200 nucleotides without protein-coding ability [5]. It has been addressed that many lncRNAs may play a pivotal role in tumor carcinogenesis. For instance, Xiong et al. pointed out that lncRNA MYOSLID could promote the invasion and metastasis of head and neck squamous cell carcinoma via modulating epithelial-to-mesenchymal transition [6]. It was also reported that lncRNA ZFAS1 may be an oncogene in head and neck squamous cell carcinoma [7]. Besides, lncRNA AC091729.7 has been discovered as a novel lncRNA that promotes the proliferation and invasion of SNSCC cells through binding to serine/arginine-rich splicing factor 2 (SRSF2) [8]. As for nuclear-enriched abundant transcript 1 (NEAT1), Wang et al. elucidated that it could promote laryngeal squamous cell cancer through regulation of the miR-107/CDK6 pathway [9]. However, the role of NEAT1 in SNSCC requires further investigation.

Previous study showed that lncRNAs may act as competitive endogenous RNAs (ceRNAs) of microRNAs (miRNAs; miRs), that is, lncRNAs may ‘sponge’ miRNAs to inhibit miRNA functions by competitively binding to microRNA response elements (MREs) [10]. Among them, NEAT1 was found to promote the progression of aggressive endometrial cancer cells via a network mediated by miR-361 [11]. It was also reported to play a key role in sepsis-induced acute kidney injury via targeting miR-204 and modulating the NF-κB pathway [12]. In addition, NEAT1 could target miR-34a-5p and promoted the progression of nasopharyngeal carcinoma [13]. Vascular endothelial growth factor A (VEGFA), a member of the VEGF family, has been found implicated in the progression of many human malignancies [14]. However, the molecular mechanisms of NEAT1 and its correlation with miR-195-5p and VEGFA in SNSCC remain inadequately discussed. Therefore, the present study aimed to discover the correlations among NEAT1, miR-195-5p and VEGFA by unveiling the molecular mechanisms via which NEAT1 played a role in SNSCC, so as to determine their roles in SNSCC and find a possible therapeutic method for SNSCC.

## Materials and methods

### Clinical samples

In the present study, tumor tissues from patients diagnosed with SNSCC (*n*=30) and turbinate mucosal tissues from normal healthy patients (*n*=20) were collected from Affiliated Hospital of Chengde Medical University between June 2019 and December 2019. All patients enrolled met the following criteria: (a) the patients had not received chemotherapy or radiotherapy treatment; (b) the patients had no other cancers, autoimmune diseases, contagious diseases or other diseases. Clinical samples were available at the initial resection and preserved in a refrigerator at −80°C after the tissues were washed with phosphate buffered saline (PBS).

### Cell culture

Human SNSCC RPMI-2650 cells (catalog no. CCL-30) were purchased from the American Type Culture Collection (ATCC; Manassas, VA, U.S.A.) and maintained in minimum essential medium (MEM; Avantor, Radnor, PA, U.S.A.) supplemented with 0.5% fetal bovine serum (FBS; S11150, R&D Systems, Minneapolis, MI, U.S.A.), 1% non-essential amino acids (NEAA) and 1% l-Glutamine (G7513; Sigma–Aldrich, St. Louis, MO, U.S.A.) in a humidified incubator at 37°C with 5% CO_2_.

For capillary-like tube formation assay, human umbilical vein endothelial cells (HUVECs) were also obtained from ATCC (catalog number: PCS-100-010) and then cultured in endothelial cell medium-2 (ECM; ScienCell, Carlsbad, CA, U.S.A.) supplemented with 5% FBS (R&D Systems, U.S.A.), endothelial cell growth supplement (ECGS; #1062, ScienCell, U.S.A.), 100 U/ml penicillin and 100 mg/ml streptomycin (Invitrogen, Carlsbad, CA, U.S.A.) in an incubator at 37°C with 5% CO_2_.

### Transfection

To investigate the effects of lncRNA NEAT1 and miR-195-5p on RPMI-2650 cells, RPMI-2650 cell transfection was performed according to the manufacturer’s instructions. For transfection, RPMI-2650 cells were cultured in six-well plates at 1 × 10^6^ cells/well. A hundred pmol of small interfering RNA for NEAT1 (siNEAT1) and VEGFA (siVEGFA) or siRNA negative control (siNC) were purchased from Invitrogen, whereas miR-195-5p mimic and inhibitor as well as their controls were obtained from GenePharma (Shanghai, China). Overexpression plasmids of VEGFA were successfully constructed with pcDNA3.1 plasmid (V79020; Thermo Fisher Scientific, Waltham, MA, U.S.A.). Transfection was performed with Lipofectamine (LFN) 3000 reagent (Invitrogen, U.S.A.) following the protocol of the manufacturer. Sequences for transfection are listed in Table 1. Cells were harvested 48 h after transfection for subsequent study and the transfection rate was measured by quantitative real-time polymerase chain reaction (qRT-PCR).

**Table 1:** Sequence for transfection

Gene	Sequence
miR-195-5p mimic	5′-UAGCAGCACAGAAAUAUUGGC-3′
miR-195-5p inhibitor	5′-GCCAAUAUUUCUGUGCUGCUA-3′
miR-195-5p mimic control	5′-UUCUCCGAACGUGUCACGUUU-3′
miR-195-5p inhibitor control	5′-AAACGUGACACGUUCGGAGAA-3′
siNEAT1	5′-UAGAGAAAAGUCCAAAAGGAG-3′
siVEGFA	5′-AAUGAAUAUCAAAUUCCAGCA-3′

### Target gene prediction and dual-luciferase reporter assay

Using StarBase and TargetScan, we successfully predicted the target genes and potential binding sites of NEAT1, miR-195-5p and VEGFA, which were then confirmed by dual-luciferase reporter assay.

The 3′UTRs of NEAT1 and VEGFA harboring miR-195-5p target sites were synthesized by Gene Pharma (Shanghai, China) and inserted into the pMirGLO luciferase vector (AM5795; Thermo Fisher Scientific, U.S.A.) to form wild-type NEAT1 and VEGFA (NEAT1-wt; VEGFA-wt) reporter plasmids. A site-directed mutagenesis kit (F541; Thermo Fisher Scientific, U.S.A.) was used to perform 3′UTR mutagenesis at the miR-195-5p target site so as to form mutated NEAT1 and VEGFA (NEAT1-mut; VEGFA-mut) reporter plasmids.

Dual-luciferase reporter assay was then performed to confirm the target gene and potential binding sites. In brief, RPMI-2650 cells were first cultured in a 96-well plate at an adjusted density of 5 × 10^3^ cells/well, and then transfected with 200 ng of miR-195-5p mimic or mimic control, and 50 ng of recombinant reporter plasmids containing wild-type or mutated NEAT1 and VEGFA (NEAT1-WT, sequence: 5′-CUGUGGACUGCUUGCUGCUU-3′; NEAT1-MUT, sequence: 5′-CUGUGGCCGGAGGAUGUAGU-3′; VEGFA-WT, sequence: 5′-CCAUUUUAUUUUUCUUGCUGCUA-3′; VEGFA-MUT, sequence: 5′-CCAUUUUUAUUUUUCUGAUCAAGA-3′) using Lipofectamine 3000 reagent (Thermo Fisher Scientific, U.S.A.). For luciferase detection, RPMI-2650 cells were harvested at 48 h after transfection, and luciferase activity was detected with dual-luciferase reporter assay system (E1910; Promega, Madison, MI, U.S.A.). Firefly luciferase activity was normalized to *Renilla* luciferase activity.

### MTT assay

RPMI-2650 cells (1 × 10^5^ cells/ml) were seeded in 96-well plates and then added with 10 μl of MTT solution (#30006; Biotium, Beijing, China). After incubation at 37°C for 4 h, 100 μl of dimethyl sulfoxide (DMSO; 472301, Sigma–Aldrich, U.S.A.) was added to dissolve formazan salt crystals. OD values at 490 nm were measured and recorded using an HTX Multi-Mode microplate reader (Catalog No. BTS1LFTA, BioTek™, Winooski, VT, U.S.A.).

### Flow cytometry

After transfection for 48 h, 1 × 10^5^ RPMI-2650 cells were treated with 5 μl of Annexin V and 5 μl of propidium iodide (PI) for 15 min in the dark at room temperature. Cell apoptosis was detected using an Annexin V-FITC cell apoptosis kit (130-092-052; Miltenyi Biotech, Waltham, MA, U.S.A.) and data were analyzed using Kaluza C Analysis Software (Beckman Coulter, Indianapolis, IN, U.S.A.).

### Capillary-like tube formation assay

Capillary-like tube formation assay was performed as previously described [15]. In detail, after being cultured alone for 6–8 h, HUVECs were co-cultured with RPMI-2650 cells (2 ×10^4^ cell/well) in a 96-well plate. The cells were then plated on pre-chilled Matrigel (50 μl; BD Biosciences, Franklin Lakes, NJ, U.S.A.) in MEM at 37°C for 1 h. Next, the plate containing the medium was exposed to Niclosamide (5 μM; N3510; Sigma–Aldrich, U.S.A.) for 8 h. Photos of tubular structures were taken and observed using an optical microscope with a recording camera (DP27; Olympus, Tokyo, Japan). Five fields were randomly selected from each well for evaluation of tube formation, and the data were further analyzed using Tube Formation ACAS Image Analysis Software (v.1.0, ibidi GmbH, Gräfelfing, Germany).

### RNA isolation and qRT-PCR

Total RNA from SNSCC tissues and cells was extracted with TRIzol reagent (A33250, Invitrogen, U.S.A.) in accordance with the manuals of the manufacturer, and then preserved in a −80°C refrigerator. Concentration of the total RNA was quantified using a biological spectrometer (NanoDrop 2000, Thermo Fisher Scientific, U.S.A.). One microgram of the total RNA was synthesized into cDNA using a First-strand cDNA Synthesis Kit (04379012001; Roche Life Sciences, Mannheim, Germany) following the manufacturer’s manuals. Then the qRT-PCR experiment was conducted using a qScript One-Step RT-qPCR kit (95057-050, Quanta Bio, Beverly, MA, U.S.A.) in real-time PCR Detection system (LineGene 9600 Plus; Biosan; Riga, Latvia) under the following conditions: at 95°C for 10 min, followed by 40 cycles at 95°C for 10 s, at 60°C for 15 s and at 72°C for 10 s. Primer sequences used in this experiment are listed in Table 2. GAPDH and U6 were used as internal controls. Expressions of relative genes were quantified by the 2^−ΔΔ*C*_T_^ calculation method [16].

**Table 2:** Primers for qRT-PCR

Gene	Primers
*miR-195-5p*	
Forward	5′-GGCGTCGTATCCAGTGCAAT-3′
Reverse	5′-GTCGTATCCAGTGCGTGTCG-3′
*NEAT1*	
Forward	5′-GGAAGGCAGGGAGAGGTAGA-3′
Reverse	5′-GGGTTCACAGCCCTTGGT-3′
*VEGFA*	
Forward	5′-GAATGGGGAGCCCAGAGT-3′
Reverse	5′-CCACTTCGTGATGATTCTGC-3′
*U6*	
Forward	5′- CTCGCTTCGGCAGCACA-3′
Reverse	5′- AACGCTTCACGAATTTGCGT-3′
*β-actin*	
Forward	5′-ACCCACACTGTGCCCATCTAC-3′
Reverse	5′- TCGGTGAGGATCTTCATGAGGTA-3′

### Western blot

In our study, Western blot was applied to measure protein expressions of related mRNAs as previously described [17]. Protein was lysed and extracted with RIPA Lysis and Extraction buffer (786-489; G-Biosciences, St. Louis, MO, U.S.A.) after the cells were collected, and its concentration was measured using a bicinchoninic acid (BCA) protein kit (K813; BioVision, Milpitas, CA, U.S.A.). Then sample lysates of protein (30 μg) were electrophoresed by 12% sodium dodecyl sulfate/polyacrylamide gel electrophoresis (SDS/PAGE; P0012A; Beyotime, Shanghai, China) and transferred into Immun-Blot® polyvinylidene fluoride (PVDF) membrane (1620174; Bio-Rad, U.S.A.). The membrane was subsequently blocked using skimmed milk (5%) for 2 h and incubated with primary antibodies: anti-VEGFA antibody (rabbit, 1:10000, ab52917, Abcam, Cambridge, U.K.), anti-phosphorylated PI3K (p-PI3K; PI3K, phosphatidylinositide 3-kinase) antibody (rabbit, 1:1000, ab182651, Abcam, U.K.), anti-PI3K antibody (mouse, 1:1000, ab86714, Abcam, U.K.), anti-phosphorylated AKT (p-AKT; AKT, Protein Kinase B) antibody (rabbit, 1:2000, #4060, Cell Signaling Technology, Danvers, MA, U.S.A.), anti-AKT antibody (rabbit, 1:1000, #4691, Cell Signaling Technology, U.S.A.) and anti-β-actin antibody (rabbit, 1:10000, ab181602, Abcam, U.K.) at 4°C overnight, with β-actin as the internal control. Then the membrane was incubated with the horseradish peroxidase (HRP)-conjugated secondary antibodies: goat anti-rabbit IgG H&L (HRP) (1:10000, sc-2004, Santa Cruz Biotechnology, Dallas, TX, U.S.A.) and goat anti-rabbit IgG H&L (HRP) (1:10000, sc-2005, Santa Cruz Biotechnology, U.S.A.) at room temperature for 1 h, and thereafter washed with Tris-buffer saline Tween (TBST) for three times. Protein band was then collected from samples and analyzed using an Amersham enhanced chemiluminescence (ECL) Western Blotting kit (RPN2108; Global Life Sciences Solutions, Pittsburgh, PA, U.S.A.). The gray values of the strips were further gathered and calculated using ImageJ 5.0 (National Institutes of Health, Bethesda, MD, U.S.A.).

### Statistical analysis

Experiments in our study were performed at least three times independently. Data were expressed as mean ± standard deviation (SD). Analysis of the statistics was performed using SPSS 21.0 software (IBM Corporation, Armonk, NY, U.S.A.). Statistical significance was assessed by one-way ANOVA and Student’s *t* test followed by Dunnett’s post hoc test. Correlation analysis of NEAT1, miR-195-5p and VEGFA was performed by Pearson’s correlation test. *P*<0.05 was considered statistically significant.

## Results

### MiR-195-5p could competitively bind to NEAT1 and target VEGFA

**Figure 1:**
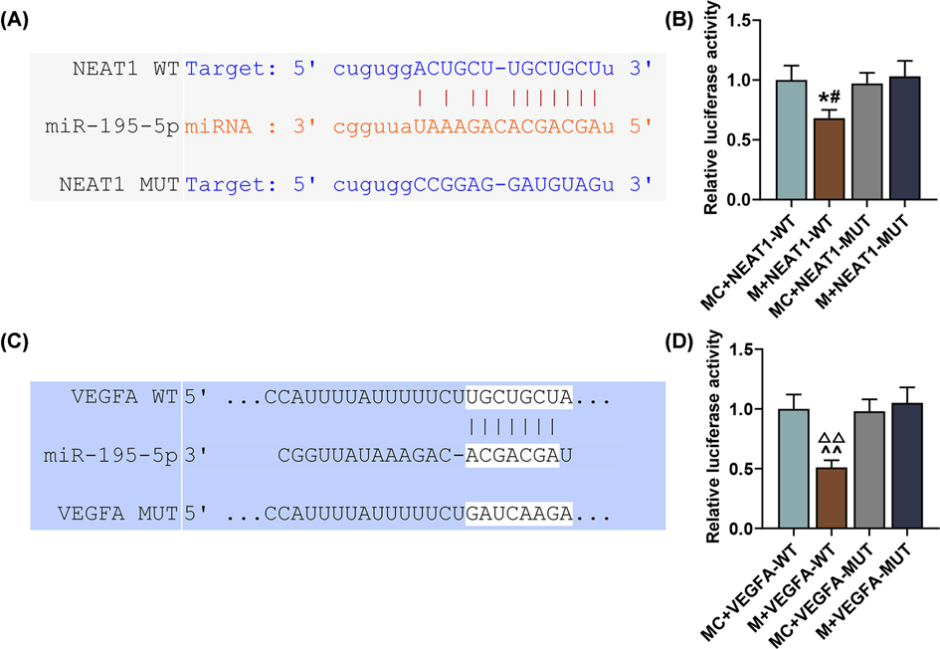
miR-195-5p could competitively bind to NEAT1 and target VEGFA (**A**) Target gene and binding sites of NEAT1 and miR-195-5p at 3′-untranslated region (UTR) and the wild-type and mutated 3′-UTRs of NEAT1 (NEAT1 3′-UTR WT; NEAT1 3′-UTR MUT) are shown. (**B**) Dual-luciferase reporter assay confirmed that miR-195-5p could competitively bind to NEAT1. (**C**) Target gene and binding sites of miR-195-5p and VEGFA at 3′-UTR and the wild-type and mutated 3′-UTR of VEGFA (VEGFA 3′-UTR WT; VEGFA 3′-UTR MUT) are shown. (**D**) Dual-luciferase reporter assay confirmed that VEGFA was the target gene of miR-195-5p. All experiments were performed in triplicate and experimental data were expressed as mean ± SD. ^#^*P*<0.05, vs. M+NEAT1-MUT; *P<0.05, vs. MC+NEAT1-WT; ^∧∧^*P*<0.01, vs. MC+VEGFA-WT; ^ΔΔ^*P*<0.01, vs. M+VEGFA-MUT. Abbreviations: M, mimic; MC, mimic control.

Using StarBase and TargetScan, we successfully recognized miR-195-5p as the candidate miRNA which could not only competitively bind to NEAT1 but also target VEGFA. The complementary binding sites are listed in Figure 1A,C. Then, for dual-luciferase reporter assay, wild-type or mutated NEAT1 and VEGFA (NEAT1-WT; NEAT1-MUT; VEGFA-WT; VEGFA-MUT) as well as miR-195-5p mimic or mimic control were co-transfected into RPMI-2650 cells. It was found that the luciferase activity in PRMI-2650 cells of the M+NEAT1-WT group was down-regulated as compared with the MC+NEAT1-WT and M+NEAT1-MUT groups (Figure 1B, *P*<0.05), whereas that in the M+NEAT1-MUT group was not affected. The same result was found in the M+VEGFA-WT group as compared with the MC+VEGFA-WT and M+VEGFA-MUT groups (Figure 1D, *P*<0.01). These results suggested that miR-195-5p could bind to NEAT1 and target VEGFA.

### Expressions and correlation analysis of NEAT1, VEGFA and miR-195-5p in SNSCC

**Figure 2:**
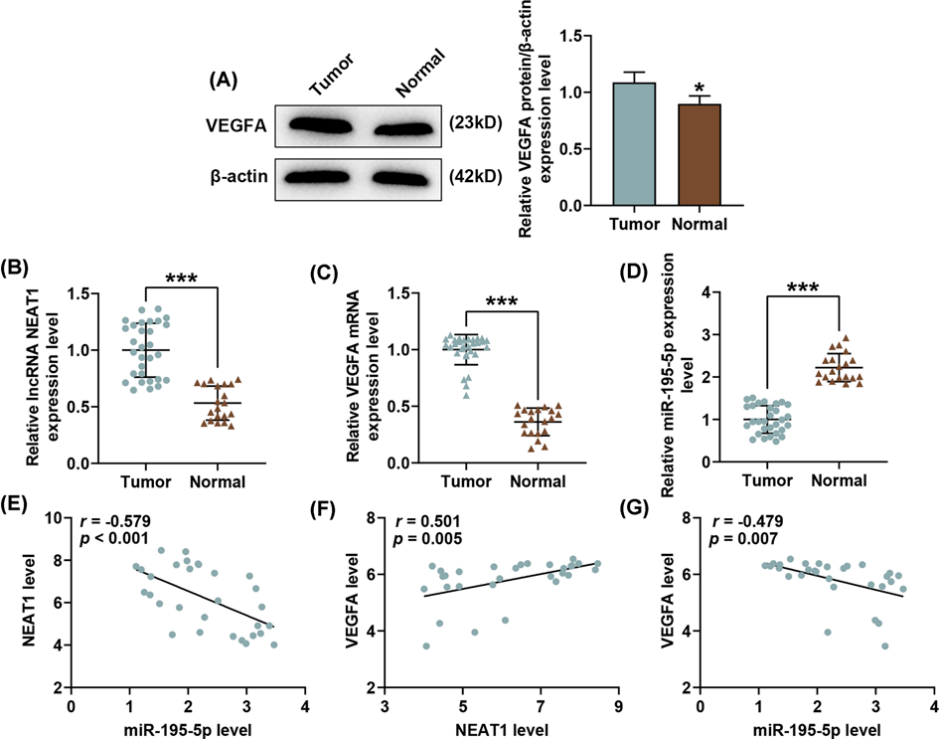
Expressions and correlation analysis of NEAT1, VEGFA and miR-195-5p in SNSCC (**A**) Relative protein/β-actin expressions of VEGFA in SNSCC tissues (Tumor, *n*=30) and healthy tissues (Normal, *n*=20) were measured by Western blot. β-actin was employed as the internal control. (**B**–**D**) Relative NEAT1, VEGFA and miR-195-5p expressions in SNSCC tissues (Tumor, *n*=30) and healthy tissues (Normal, *n*=20) were measured by qRT-PCR. β-actin (for NEAT1 and VEGFA) and U6 (for miR-195-5p) were used as internal controls. (**E–G**) Correlation analysis of NEAT1, miR-195-5p and VEGFA was performed by Pearson’s correlation test. All experiments were performed in triplicate and experimental data were expressed as mean ± SD. *P<0.05, ***P<0.001, vs. Tumor.

In order to further explore the roles of NEAT1, miR-195-5p and VEGFA in SNSCC, we measured their expressions in tissues of patients diagnosed with SNSCC (*n*=30) and turbinate mucosal tissues from normal healthy patients (*n*=20). Western blot and qRT-PCR results in Figure 2A–D demonstrated that both NEAT1 and VEGFA expressions were up-regulated yet miR-195-5p expression was down-regulated in SNSCC tissues (Tumor) as compared with healthy tissues (Normal) (*P*<0.001). Also, the results from Pearson’s correlation test showed that in SNSCC, NEAT1 was negatively correlated with miR-195-5p (Figure 2E, *r* = −0.579, *P*<0.001) yet positively correlated with VEGFA (Figure 2F, *r* = 0.501, *P*=0.005). Furthermore, we also found a negative correlation between miR-195-5p and VEGFA (Figure 2G, *r* = −0.479, *P*=0.007).

### Effects of silenced or overexpressed VEGFA on SNSCC cell viability, apoptosis and capillary-like tube formation

**Figure 3:**
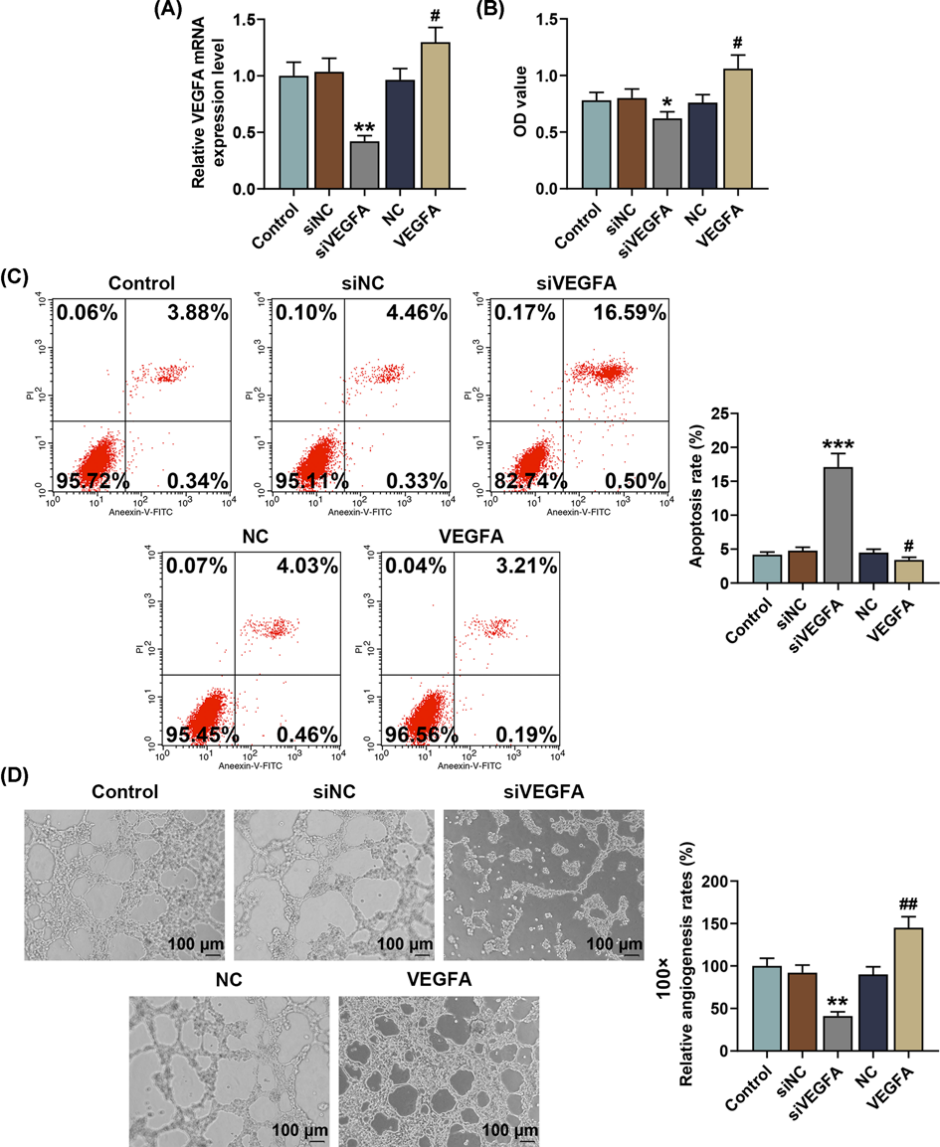
Effects of silenced and overexpressed VEGFA on VEGFA expression in SNSCC cells, and SNSCC cell viability, apoptosis and capillary-like tube formation (**A**) Relative VEGFA expressions after transfection with silenced or overexpressed VEGFA were measured by qRT-PCR. β-actin was employed as the internal control. (**B**) Relative SNSCC cell viability after transfection with silenced or overexpressed VEGFA was measured by MTT assay. (**C**) Relative apoptosis rate of SNSCC cells after tranfection with silenced or overexpressed VEGFA was measured by flow cytometry. (**D**) Relative angiogenesis rate of SNSCC cell after tranfection with silenced or overexpressed VEGFA was determined by capillary-like tube formation assay. All experiments were performed in triplicate and experimental data were expressed as mean ± SD. *P<0.05, **P<0.01, ***P<0.001, vs. siNC; ^#^*P*<0.05, ^##^*P*<0.01, vs. NC.

To discover the role of VEGFA in SNSCC cells, we transfected VEGFA overexpression plasmid as well as small interfering RNA for VEGFA (siVEGFA) into SNSCC RPMI-2650 cells. As shown in Figure 3A, VEGFA expression was down-regulated after transfection with siVEGFA, whereas transfection with the VEGFA overexpression plasmid led to an opposite effect (*P*<0.01), suggesting that overexpressed VEGFA up-regulated VEGFA expression in SNSCC cells, whereas silencing VEGFA caused an opposite effect.

We then detected the effects of overexpressed or silenced VEGFA on SNSCC cell viability, apoptosis and vascular mimicry (VM) formation by MTT assay, flow cytometry and capillary-like tube formation assay, respectively. As shown in Figure 3B, there was a decrease in the viability of SNSCC cells after silencing VEGFA, whereas overexpressed VEGFA resulted in an opposite effect (*P*<0.05), indicating that silencing VEGFA suppressed SNSCC cell viability while overexpressed VEGFA exerted an opposite effect.

It was discovered from the results of flow cytometry that after silencing VEGFA, the apoptosis rate of SNSCC cells was significantly up-regulated while overexpressed VEGFA caused a decrease in the apoptosis rate of SNSCC cells (Figure 3C, *P*<0.001), suggesting that silencing VEGFA resulted in the promotion of SNSCC cell apoptosis, whereas overexpressed VEGFA posed an opposite effect.

According to the results from capillary-like tube formation assay in Figure 3D, the relative angiogenesis rate of HUVECs mediated by SNSCC cells in the siVEGFA group was decreased (*P*<0.01). Overexpressed VEGFA, however, resulted in a contrary result (Figure 3D, *P*<0.01). Therefore, it could be summarized that silencing VEGFA suppressed capillary-like tube formation in SNSCC cell-mediated HUVECs whereas overexpressed VEGFA led to a contrary result.

### Down-regulating miR-195-5p reversed the effects of silencing NEAT1 on NEAT1 and miR-195-5p expressions, and SNSCC cell viability, apoptosis and capillary-like tube formation

**Figure 4:**
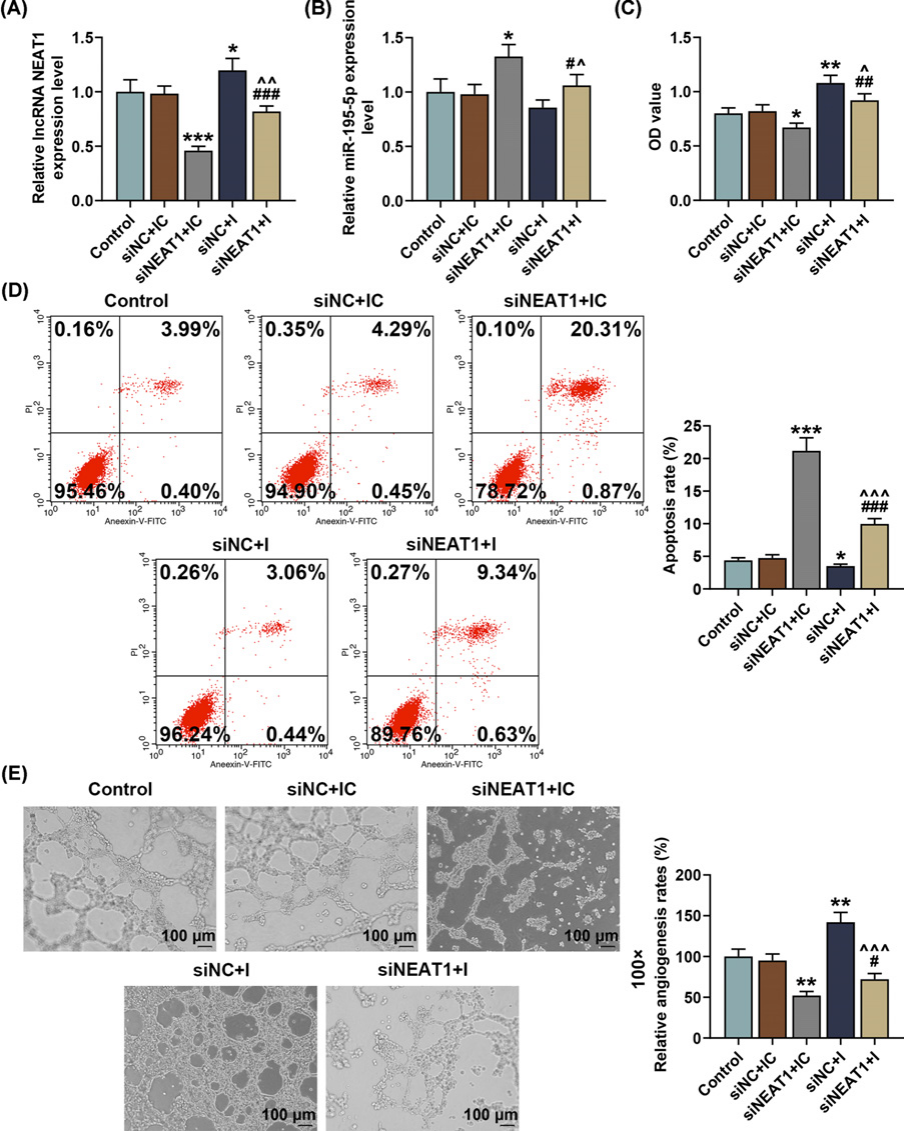
Down-regulating miR-195-5p reversed the effects of silencing NEAT1 on NEAT1 and miR-195-5p expressions and SNSCC cell behavior (**A,B**) Relative NEAT1 and miR-195-5p expressions after silencing NEAT1 and down-regulating miR-195-5p were measured by qRT-PCR. β-actin (for NEAT1) and U6 (for miR-195-5p) were employed as internal controls. (**C**) Relative SNSCC cell viability after silencing NEAT1 and down-regulating miR-195-5p was detected by MTT assay. (**D**) Relative apoptosis rate of SNSCC cells after silencing NEAT1 and down-regulating miR-195-5p was measured by flow cytometry. (**E**) Relative angiogenesis rate of SNSCC cells after silencing NEAT1 and down-regulating miR-195-5p was measured by capillary-like tube formation assay. All experiments were performed in triplicate and experimental data were expressed as mean ± SD. *P<0.05, **P<0.01, ***P<0.001, vs. siNC+IC; ^#^*P*<0.05, ^##^*P*<0.01, ^###^*P*<0.001, vs. siNEAT1+NC; ^∧^*P*<0.05, ^∧∧^*P*<0.01, ^∧∧∧^*P*<0.001, vs. siNC+I.

To find out the role of NEAT1 and its correlation with miR-195-5p in SNSCC cells, we transfected siNEAT1 as well as miR-195-5p inhibitor into SNSCC RPMI-2650 cells. As shown in Figure 4A,B, NEAT1 expression was down-regulated yet miR-195-5p expression was increased after transfection with siNEAT1, whereas transfection with miR-195-5p inhibitor led to an opposite effect (*P*<0.01). Furthermore, we also found that the effects of silencing NEAT1 on NEAT1 and miR-195-5p expressions in SNSCC cells were reversed by down-regulating miR-195-5p (Figure 4A,B, *P*<0.001).

Using MTT assay, flow cytometry and capillary-like tube formation assay, we then detected the effects of NEAT1 and miR-195-5p on SNSCC cell behavior. As shown in Figure 4C, the results from MTT assay showed that silencing NEAT1 caused a decrease in SNSCC cell viability, whereas down-regulating miR-195-5p resulted in an opposite effect (*P*<0.05). In addition, we discovered that down-regulating miR-195-5p could reverse the effects of silencing NEAT1 on SNSCC cell viability (Figure 4C, *P*<0.01).

Flow cytometry results revealed that after silencing NEAT1, the apoptosis rate of SNSCC cells was significantly up-regulated, while down-regulating miR-195-5p led to an opposite effect (Figure 4D, *P*<0.05). Besides, down-regulating miR-195-5p was found to reverse the effects of silencing NEAT1 on SNSCC cell apoptosis (Figure 4D, *P*<0.001).

Results from capillary-like tube formation exhibited a decrease in the angiogenesis rate of SNSCC cells-mediated HUVECs after silencing NEAT1, whereas down-regulating miR-195-5p caused an opposite effect (Figure 4E, *P*<0.01). In addition, we discovered that down-regulating miR-195-5p could reverse the effects of silencing NEAT1 on capillary-like tube formation of SNSCC cells-mediated HUVECs (Figure 4E, *P*<0.05).

### Down-regulating miR-195-5p reversed the effects of silencing NEAT1 on VEGFA, p-PI3K and p-AKT expressions in SNSCC cells

**Figure 5:**
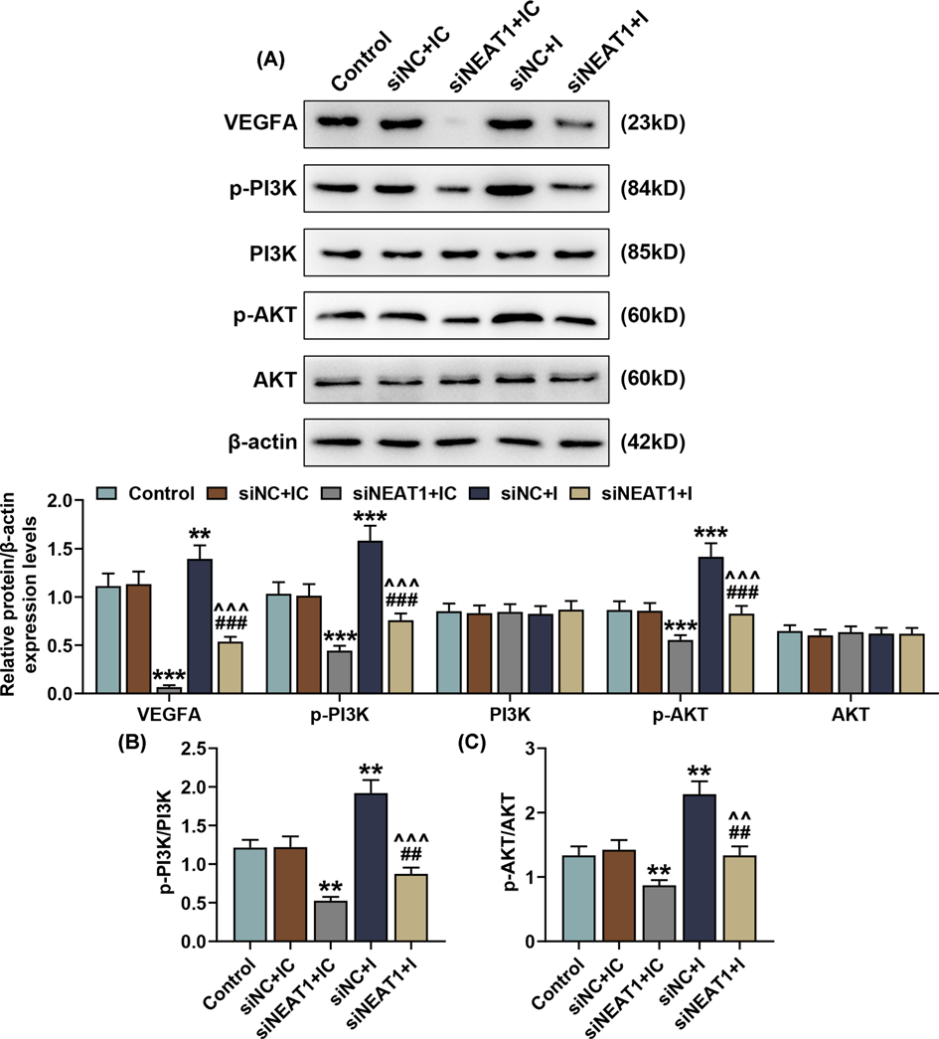
Down-regulating miR-195-5p reversed the effects of silencing NEAT1 on VEGFA, p-PI3K and p-AKT expressions in SNSCC cells (**A**) VEGFA, p-PI3K and p-AKT protein/β-actin expressions in SNSCC cells after silencing NEAT1 and down-regulating miR-195-5p were measured by Western blot. β-actin was employed as the internal control. (**B,C**) Ratios of p-PI3K/PI3K and p-AKT/AKT were calculated. All experiments were performed in triplicate and experimental data were expressed as mean ± SD. **P<0.01, ***P<0.001, vs. siNC+IC; ^##^*P*<0.01, ^###^*P*<0.001, vs. siNEAT1+NC; ^∧∧^*P*<0.01, ^∧∧∧^*P*<0.001, vs. siNC+I. Abbreviations: I, inhibitor; IC, inhibitor control.

To discover the effects of NEAT1 and miR-195-5p on VEGFA, PI3K and AKT expressions in SNSCC cells, we measured these expressions in SNSCC cells after silencing NEAT1 and down-regulating miR-195-5p by Western blot. As shown in Figure 5A, VEGFA, p-PI3K and p-AKT expressions were decreased after silencing NEAT1, whereas down-regulating miR-195-5p resulted in a contrary result (*P*<0.001). We also found that the effects of silencing NEAT1 on VEGFA, p-PI3K and p-AKT expressions in SNSCC cells were reversed by down-regulating miR-195-5p (Figure 5A, *P*<0.01).

Furthermore, we verified PI3K and AKT phosphorylation in SNSCCs after silencing NEAT1 and down-regulating miR-195-5p. In this section, we found that silencing NEAT1 evidently down-regulated the phosphorylation levels of PI3K and AKT in SNSCCs (Figure 5B,C, *P*<0.01). However, an opposite result was obtained after down-regulating miR-195-5p, which suggested that down-regulating miR-195-5p could enhance the levels of PI3K and AKT phosphorylation in SNSCCs (Figure 5B,C, *P*<0.01). In conclusion, down-regulating miR-195-5p reversed the effects of silencing NEAT1 on the phosphorylation levels of PI3K and AKT in SNSCC cells.

### Overexpressed VEGFA reversed the effects of miR-195-5p up-regulation on VEGFA and miR-195-5p expressions in SNSCC cells

**Figure 6:**
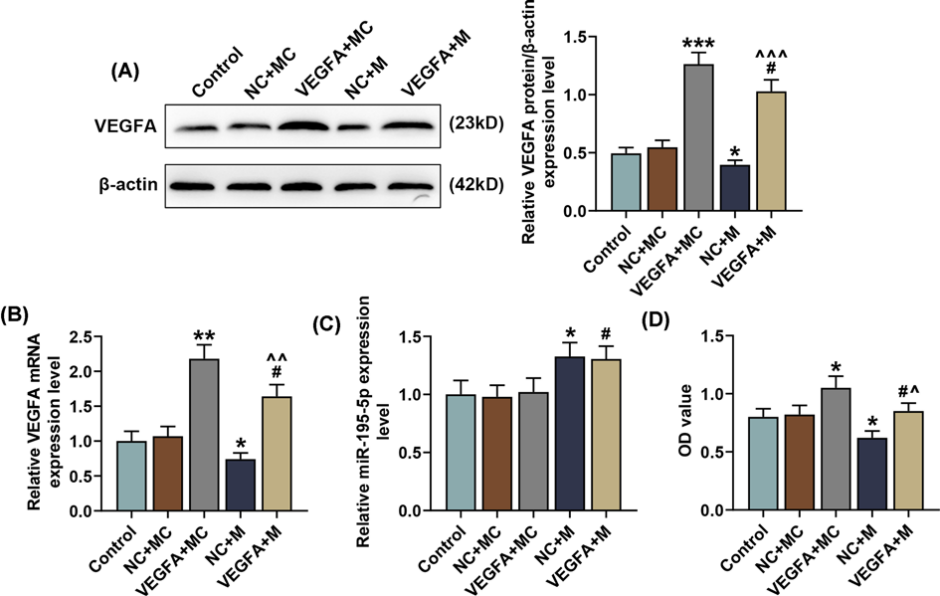
Overexpressed VEGFA reversed the effects of miR-195-5p up-regulation on VEGFA expression and SNSCC cell viability (**A,B**) Relative expressions of VEGFA protein/β-actin and mRNA in SNSCC cells after transfection with the VEGFA overexpression plasmid and up-regulating miR-195-5p expression were measured by Western blot and qRT-PCR. β-actin was employed as the internal control. (**C**) Relative miR-195-5p expressions in SNSCC cells after transfection with the VEGFA overexpression plasmid and up-regulating miR-195-5p expression were measured by qRT-PCR. U6 was chosen as the internal control. (**D**) SNSCC cell viability after transfection with the VEGFA overexpression plasmid and up-regulating miR-195-5p expression was measured by MTT assay. All experiments were performed in triplicate and experimental data were expressed as mean ± SD. *P<0.05, **P<0.01, ***P<0.001, vs. NC+MC; ^#^*P*<0.05, vs. VEGFA+MC; ^∧^*P*<0.05, ^∧∧^*P*<0.01, ^∧∧∧^*P*<0.001, vs. NC+M. Abbreviation: MC, mimic control.

To find out the role of miR-195-5p and its correlation with VEGFA in SNSCC cells, we transfected miR-195-5p mimic and the VEGFA overexpression plasmid into SNSCC RPMI-2650 cells. As shown in Figure 6A–C, overexpressed VEGFA increased the protein and mRNA expressions of VEGFA (*P*<0.01) yet had no significant effect on miR-195-5p expression, while transfection with miR-195-5p mimic led to an opposite effect (*P*<0.05). Furthermore, we also found that overexpressed VEGFA reversed the effects of up-regulating miR-195-5p on VEGFA and miR-195-5p expressions (Figure 6A–C, *P*<0.05).

### Overexpressed VEGFA reversed the effects of miR-195-5p up-regulation on SNSCC cell viability, apoptosis and capillary-like tube formation

Then, we detected the effects of VEGFA and miR-195-5p on SNSCC cell behavior by MTT assay, flow cytometry and capillary-like tube formation assay. As shown in Figure 6D, the results from MTT assay exhibited an increase in SNSCC cell viability after transfection with the VEGFA overexpression plasmid, whereas up-regulating miR-195-5p resulted in an opposite effect (*P*<0.05). In addition, we discovered that the effects of up-regulating miR-195-5p on SNSCC cell viability were reversed by overexpressed VEGFA (Figure 6D, *P*<0.01).

**Figure 7:**
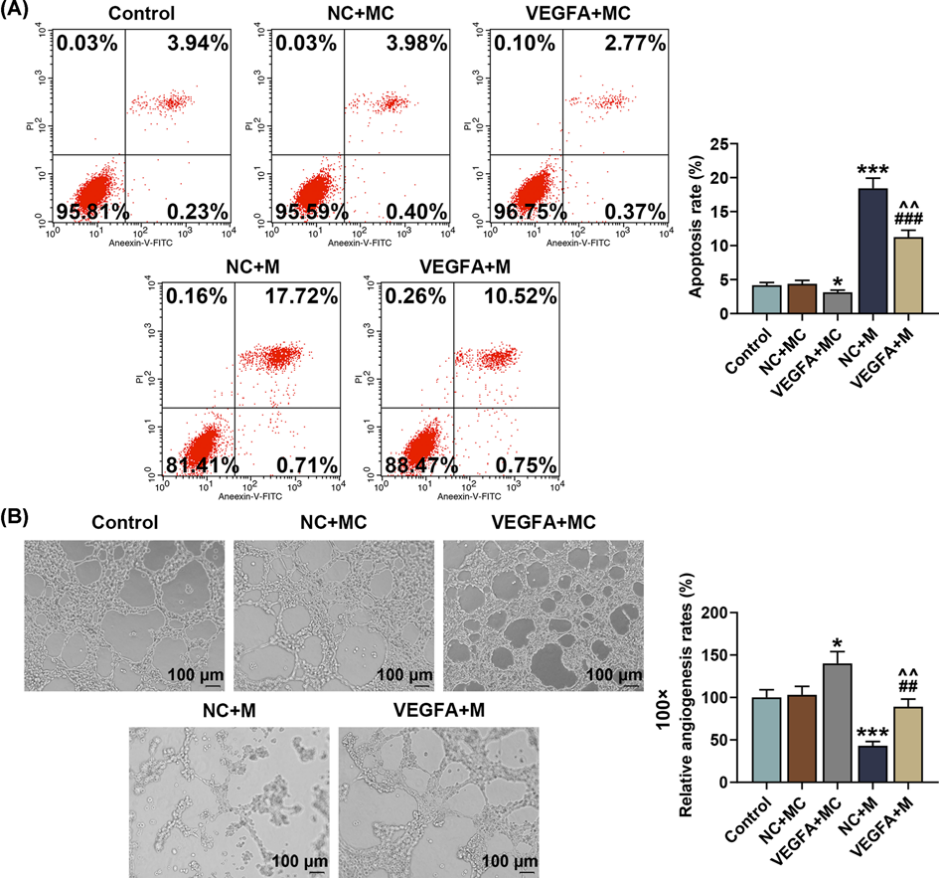
Overexpressed VEGFA reversed the effects of miR-195-5p up-regulation on VEGFA expression and cell viability in SNSCC cells (**A**) Apoptosis rate of SNSCC cells after transfection with overexpressed VEGFA plasmid and up-regulating miR-195-5p expression was measured by flow cytometry. (**B**) Angiogenesis rate of SNSCC cells after transfection with overexpressed VEGFA plasmid and up-regulating miR-195-5p expression was detected by capillary-like tube formation assay. All experiments have been performed in triplicate and experimental data were expressed as mean ± SD. *P<0.05, ***P<0.001, vs. NC+MC; ^##^*P*<0.01, ^###^*P*<0.001, vs. VEGFA+MC; ^∧∧^*P*<0.01, vs. NC+M.

Flow cytometry results indicated that overexpressed VEGFA exerted a significant down-regulatory effect on the apoptosis of SNSCC cells, while up-regulating miR-195-5p led to a contrary result (Figure 7A, *P*<0.05). Besides, VEGFA overexpression was found to reverse the effects of miR-195-5p up-regulation on SNSCC cell apoptosis (Figure 7A, *P*<0.01).

Through capillary-like tube formation assay, we observed an increased angiogenesis rate of SNSCC cell-mediated HUVECs after transfection with the VEGFA overexpression plasmid, whereas up-regulating miR-195-5p caused an opposite effect (Figure 7B, *P*<0.05). In addition, we discovered that overexpressed VEGFA could reverse the effects of up-regulating miR-195-5p on capillary-like tube formation of SNSCC cell-mediated HUVECs (Figure 7B, *P*<0.01).

### Overexpressed VEGFA reversed the effects of miR-195-5p up-regulation on p-PI3K and p-AKT expressions in SNSCC cells

**Figure 8:**
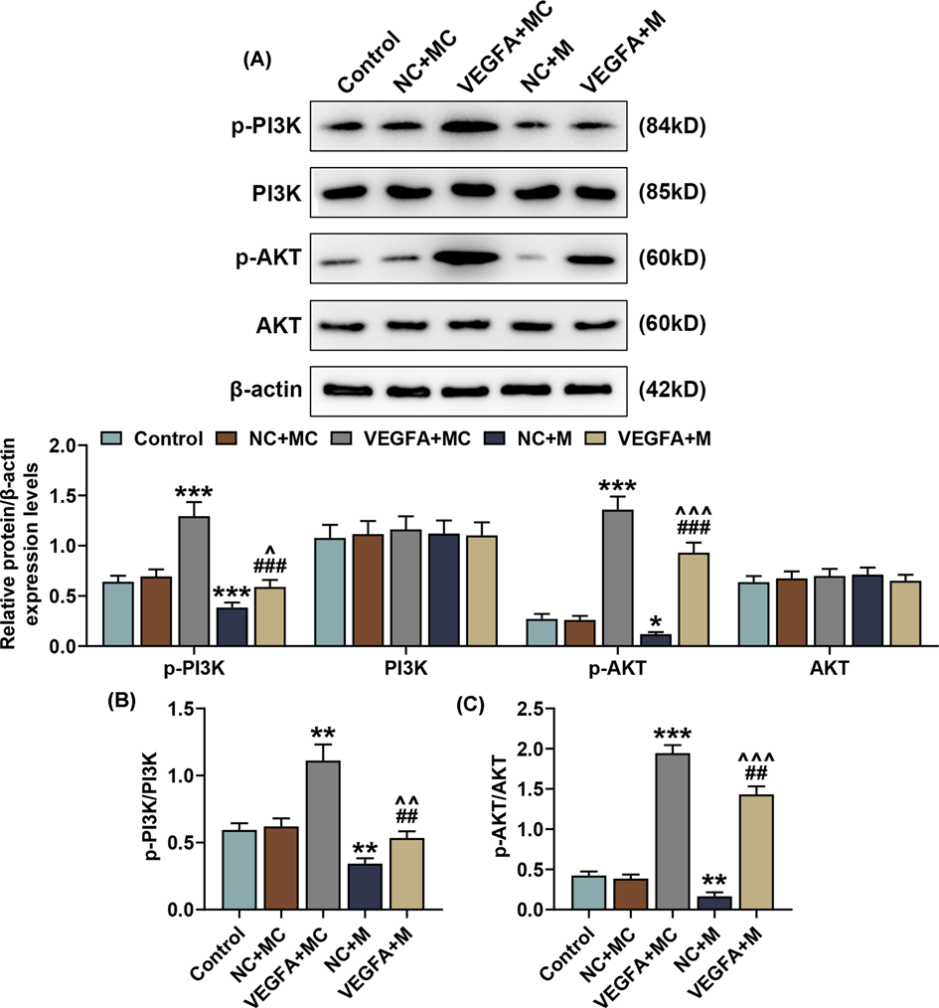
Overexpressed VEGFA reversed the effects of miR-195-5p up-regulation on the expressions and phosphorylation levels of p-PI3K and p-AKT in SNSCC cells (**A**) p-PI3K and p-AKT protein/β-actin expressions in SNSCC cells after transfection with the VEGFA overexpression plasmid and up-regulating miR-195-5p expression were measured by Western blot. β-actin was employed as the internal control. (**B,C**) Ratios of p-PI3K/PI3K and p-AKT/AKT were calculated. All experiments were performed in triplicate and experimental data were expressed as mean ± SD. *P<0.05, **P<0.01, ***P<0.001, vs. NC+MC; ^##^*P*<0.01, ^###^*P*<0.001, vs. VEGFA+MC; ^∧^*P*<0.05, ^∧∧^*P*<0.01, ^∧∧∧^*P*<0.001, vs. NC+M.

To discover the effects of VEGFA and miR-195-5p on PI3K and AKT expressions in SNSCC cells, we measured PI3K and AKT expressions in SNSCC cells after transfection with miR-195-5p mimic and the VEGFA overexpression plasmid by Western blot. As shown in Figure 8A, there was an increase in p-PI3K and p-AKT expressions after transfection with the VEGFA overexpression plasmid, whereas up-regulating miR-195-5p resulted in a contrary result (*P*<0.05). We also found that overexpressed VEGFA could reverse the effects of up-regulating miR-195-5p on p-PI3K and p-AKT expressions in SNSCC cells (Figure 8A, *P*<0.001).

Furthermore, we verified PI3K and AKT phosphorylation in SNSCCs after transfection with miR-195-5p mimic and the VEGFA overexpression plasmid. In this section, we found that overexpressed VEGFA markedly raised the phosphorylation levels of PI3K and AKT in SNSCCs (Figure 8B,C, *P*<0.01). However, an opposite result was obtained after up-regulating miR-195-5p, which suggested that up-regulating miR-195-5p could suppress the PI3K and AKT phosphorylation in SNSCCs. Also, overexpressed VEGFA could reverse the effects of up-regulating miR-195-5p on the phosphorylation levels of PI3K and AKT in SNSCC cells (Figure 8B,C, *P*<0.01).

## Discussion

Among all head and neck tumors, sinonasal malignancies rarely occur yet they are highly fatal with an overall survival rate of as low as less than 5% in patients, and therefore it is highly necessary to develop novel therapeutic target for the disease management [18]. SCC, the most common histological variant of sinonasal malignancies, has been reported to have a high incidence rate (>50%) of nasal and paranasal sinus tumors [19]. SNSCC has been found most prevalent in maxillary sinus, followed by nasal cavity. [20]. The prognosis of SNSCC patients remained poor even after surgical treatment, and therefore it is significant to further discover the molecular mechanisms related to SNSCC development and progression [21].

Multiple studies have suggested that lncRNAs might be implicated in sinonasal malignancies [22]. As an architectural component of nuclear paraspeckles, lncRNA NEAT1 has been found implicated in many human malignancies [23,24]. However, the role of NEAT1 in SNSCC development and progression remained elusive. In our present study, we found that NEAT1 could competitively bind to miR-195-5p, and NEAT1 expression was up-regulated yet miR-195-5p expression was down-regulated in SNSCC tissues. Also, the effects of silencing NEAT1 on the viability, apoptosis and capillary-like tube formation of SNSCC cells were reversed by miR-195-5p down-regulation.

The PI3K/AKT pathway has been found involved in the progression of many diseases [25], and previous studies suggested that NEAT1 could activate the PI3K/AKT pathway so as to promote disease progression. Xu et al. pointed out that NEAT1 could participate in the development of Multiple Myeloma (MM) through activating the PI3K/AKT pathway [26]. Also, Xia et al. found that the NEAT1/PI3K/AKT pathway might be implicated in sepsis-related inflammation [27]. In addition, NEAT1 down-regulation could result in the inactivation of the PI3K/AKT pathway [28]. However, the roles of NEAT1 and the PI3K/AKT pathway in SNSCC remained poorly understood. In our present study, we found that NEAT1 down-regulation could inhibit the activation of PI3K/AKT pathway and thereby inhibit SNSCC progression. However, the effects of silencing NEAT1 on the PI3K/AKT pathway were reversed by miR-195-5p down-regulation in SNSCC cells.

VM is defined as a process in which invasive tumor cells can simulate endothelial cells and form a pipeline structure, and its presence has been found associated with high tumor grade, short survival, invasion and metastasis [29]. Since first discovered in melanoma, VM has been found in multiple human malignancies, such as breast cancer [30], hepatocellular carcinoma [31] and glioma [32]. Many discoveries have brought novel insights into the molecular mechanisms governing VM, and it has been addressed that VEGFA played a pivotal role in VM formation [33]. VEGFA was found to bind to and activate two tyrosine kinase receptors, namely VEGF receptor 1 (VEGFR1) and VEGF receptor 2 (VEGFR2), which have been reported to be implicated in many signaling capacities in VM channel formation [34,35]. It was also found that VEGFA could be regulated by miRNAs in multiple human malignancies. As Li et al. pointed out, VEGFA was targeted by miR-200b and its down-regulation might contribute to the amelioration of diabetic retinopathy [36]. In colorectal cancer progression, miR-150-5p was discovered to act as a suppressor via targeting VEGFA [37]. In addition, miR-15a-5p could suppress peritoneal dialysis-induced inflammation and fibrosis of peritoneal mesothelial cells by targeting VEGFA [38]. As for miR-195, Liu et al. found that VEGF was the target of miR-195 and miR-195 could suppress the metastasis and angiogenesis of squamous cell lung cancer (SQCLC) cells [39]. However, the roles of miRNA, miR-195-5p in particular, and VEGFA in SNSCC remain 1 to be further discovered. In our present study, we found that VEGFA was the target gene of miR-195-5p, and overexpressed VEGFA reversed the effects of miR-195-5p up-regulation on the viability, apoptosis and capillary-like tube formation of SNSCC cells.

Besides, other studies suggested that VEGFA was also able to activate several signaling pathways, the PI3K/AKT pathway in particular [40]. In our present study, we found that VEGFA expression was up-regulated in SNSCC tissues, and overexpressed VEGFA promoted the viability and capillary-like tube formation yet suppressed the apoptosis of SNSCC cells, suggesting that VEGFA might also play a pivotal role in SNSCC development and progression. In addition, we found an increase in the phosphorylation levels of PI3K and AKT after transfection with the VEGFA overexpression plasmid, which therefore showed that VEGFA may promote VM formation in SNSCC via activating the PI3K/AKT signaling pathway. Moreover, overexpressed VEGFA reversed the effects of miR-195-5p up-regulation on the phosphorylation of PI3K and AKT in SNSCC cells. Besides, the inhibitory effects of silencing NEAT1 on VEGFA levels were reversed by miR-195-5p down-regulation in SNSCC cells.

However, there were some limitations to our study. In our study, the effects of NEAT1, miR-195-5p and VEGFA in SNSCC were detected mainly by experiments *in vitro*, which are yet to be validated in animal experiments. Hence, studies *in vivo* are required to further verify our results. Besides, some clinicopathological data of the SNSCC patient and healthy human samples that show the levels of NEAT1, miR-195-5p and VEGFA are also worth further study. In addition, as transcription factor NF-κB plays an important role in regulating VEGF expression, it will be interesting to observe the effect of silencing/overexpressing NEAT1 on NF-κB signaling in future study.

In conclusion, our study revealed a new role of NEAT1 in SNSCC. It was found that NEAT1 was up-regulated in SNSCC, and down-regulating NEAT1 inhibited the viability and vasculogenic mimicry formation yet promoted the apoptosis of SNSCC cell via the miR-195-5p/VEGFA axis. These results unveiled the possible molecular mechanisms of NEAT1 in SNSCC, along with the potential therapeutic target for SNSCC treatment.

## Data Availability

The analyzed datasets generated during the study are available from the corresponding author on reasonable request.

## Abbreviations

ATCC: American Type Culture Collection
CDK6: cyclin dependent kinases 6
FBS: fetal bovine serum
HRP: horseradish peroxidase
HUVEC: human umbilical vein endothelial cell
lncRNA: long non-coding RNA
MEM: minimum essential medium
miR/miRNA: microRNA
MTT: methyl thiazolyl tetrazolium
NEAT1: nuclear-enriched abundant transcript 1
OD: optical density
PI3K/AKT: phosphatidylinositide 3-kinase/Protein Kinase B
p-AKT: phosphorylated AKT
p-PI3K: phosphorylated PI3K
qRT-PCR: quantitative real-time polymerase chain reaction
SNSCC: sinonasal squamous cell carcinoma
VEGFA: vascular endothelial growth factor A
VM: vascular mimicry
